# Selection of a Medical School

**Published:** 1887-09

**Authors:** 


### Editorial.

### *SELECTION OF A MEDICAL SCHOOL.*

The time is near at hand for the student intending to study medicine to select a school. It is our duty as preceptors and instructors to give such men a word of advice as to the college where they will spend their valuable time and money, perhaps their all. It is our duty first to see that they are qualified; therefore, select a college where the *entrance examination is demanded.* No student wishes to spend two or three years in a college, attempting to learn an art which he has not the ability to understand. Therefore, if we are honest with these young men, and we perform our duty to them, we will find out if they have the ability and education to start. It is far better the student should know if he is not qualified and fit himself at once by study, or direct his attention to other fields of labor. It is a painful sight, indeed, to see young men who supposed they had a "good enough education," who have been licensed by some college, having obtained a diploma, at last realizing that they are not qualified; that their preliminary training was deficient. If they are qualified, where shall they attend? Shall they try the old and worthless plan, or the one which guarantees a successful career. If any student has doubt on this subject, let him read the following wise words from the authorities of Syracuse University, and he cannot fail to be convinced:

"There are two systems of medical education in vogue in this country. One system opens its inviting arms, without discrimination, to every applicant for medical honors, whether he be the well-trained and science-loving student, or an adventurer fresh from the forest, the anvil or the stable, whose whole literary education consists in a slight and perhaps contemptuous acquaintance with the three R's.

This system requires the student to hear lectures every day on all the branches taught, the most advanced as well as the elementary. It compels him to listen to lectures on surgery, pathology, therapeutics and obstetrics, while as yet he knows not a single syllable of anatomy, physiology and materia medica. It obliges him to waste many an hour every day of his scant first course in hearkening to what he cannot by any possibility fully comprehend, when these hours are all needed in studies which are pre-requisite. Its second, which is its final course of lectures, is a repetition of the first, with the very slightest variation; the same able actors reciting the identical parts which they rehearsed the year before.

Its first compulsory examination to test the faithfulness, ability and memory of the pupil is at the close of this twice-told course; and this examination is not infrequently so brief and superficial as to be of necessity almost worthless. The great men of the profession who were subjected to this system became eminent not as a result of this system, but in spite of it.

A better system with severe requirements would have given them a better start, and would also have excluded a horde of incompetent and dangerous pretenders from the profession.

This better system of medical education, the natural or graded one, is no longer a dreamy speculation It has been fairly tested, and it has been found efficient. Those who know it best give it the heartiest approval. Its requirements for matriculation are, or should be, more and more exacting every year.

Its written and oral examinations are frequent and thorough. Its yearly and final ordeals are very searching, but the students who have brains, application and faithfulness come through triumphantly. Even those whose natural capacity is not great succeed remarkably. Under a natural system their mental growth is rapid and astonishing. At the last they seldom fail, because their time has been husbanded and properly employed; because their studies have been interesting and delightful, inasmuch as they have been thoroughly comprehended and mastered, every step of the way.

These two systems of medical education may be contrasted briefly thus:

*In one case:* no preliminary examination; only two short courses of lectures required, the second identically with the first; the novice required to attend lectures on every subject every day, neglecting preliminary work because the time which might have been devoted to it has been worse than wasted in hearing learned talk which he was not prepared to understand; valuable clinical and laboratory opportunities wasted; no examinations to advance to higher grades, for there are no grades; a final examination which is so superficial in many instances as to be little better than a farce, and whose meshes are large enough to let mediocrity and inferiority, as well as the superiority, which is always present, pass through with greatest ease.

*In the other case:* preliminary examinations which require a certain, even if insufficient, preparation; three years of training at the college, the novice beginning at the beginning; all his time employed in hearing and doing what he can and should understand; his capability and progress tested in each department by weekly oral and monthly written examinations; his advance from grade to grade checked if his fitness be not established; thorough clinical and laboratory work; searching yearly examination; and a final examination, which, although of great severity, does not, except in rare instances, result in rejection, because the student has been educated in a way which accords with nature and does not violate it.

Of these two systems, which is likely to be the better?"

It too frequently happens that students, after they have spent their valuable time and money for a course in college, find that it is absolutely worthless, and they are obliged to drift into various trades. Some few are able to go to Europe or a post graduate school to prepare. The long and thorough course may cost more, but the money will be returned to every student who takes it, with a high rate of interest. Other reasons why we would select a college with a thorough course are, that state boards and colleges of good repute are demanding more and more of the student and new practitioners. The State Board of Illinois does not consider a college to be in good repute unless it demand an entrance examination; and in a few months it will demand that every college require three years. The College of Physicians and Surgeons will, at its next year's session, demand a rigid entrance examination. In view of these facts, no student can afford to attempt to enter a profession handicapped by a superficial education.

